# Towards automatic EEG cyclic alternating pattern analysis: a systematic review

**DOI:** 10.1007/s13534-023-00303-w

**Published:** 2023-07-19

**Authors:** Fábio Mendonça, Sheikh Shanawaz Mostafa, Fernando Morgado-Dias, Antonio G. Ravelo-García, Ivana Rosenzweig

**Affiliations:** 1 University of Madeira, Funchal, Portugal; 2 Interactive Technologies Institute (ITI/ARDITI/LARSyS), Funchal, Portugal; 3 Institute for Technological Development and Innovation in Communications, Universidad de Las Palmas de Gran Canaria, Las Palmas de Gran Canaria, Spain; 4 Sleep Disorders Centre, Guy’s and St Thomas’ NHS Foundation Trust, London, UK; 5 Sleep and Brain Plasticity Centre, Department of Neuroimaging, Institute of Psychiatry, Psychology and Neuroscience (IoPPN), King’s College London, London, UK

**Keywords:** A phase, Automatic classification, CAP, EEG

## Abstract

This study conducted a systematic review to determine the feasibility of automatic Cyclic Alternating Pattern (CAP) analysis. Specifically, this review followed the 2020 Preferred Reporting Items for Systematic reviews and Meta-Analyses (PRISMA) guidelines to address the formulated research question: is automatic CAP analysis viable for clinical application? From the identified 1,280 articles, the review included 35 studies that proposed various methods for examining CAP, including the classification of A phase, their subtypes, or the CAP cycles. Three main trends were observed over time regarding A phase classification, starting with mathematical models or features classified with a tuned threshold, followed by using conventional machine learning models and, recently, deep learning models. Regarding the CAP cycle detection, it was observed that most studies employed a finite state machine to implement the CAP scoring rules, which depended on an initial A phase classifier, stressing the importance of developing suitable A phase detection models. The assessment of A-phase subtypes has proven challenging due to various approaches used in the state-of-the-art for their detection, ranging from multiclass models to creating a model for each subtype. The review provided a positive answer to the main research question, concluding that automatic CAP analysis can be reliably performed. The main recommended research agenda involves validating the proposed methodologies on larger datasets, including more subjects with sleep-related disorders, and providing the source code for independent confirmation.

## Introduction

Sleep is a fundamental aspect of the circadian rhythm that is unique to each person and is comprised of various stages with associated autonomic nervous system activities. During sleep, the body repairs vital systems, and the sleep process significantly impacts memory consolidation, physical development, learning, emotion regulation, and overall life quality [1]. However, despite the critical role that sleep plays in maintaining physical and mental health, there remains a lack of consensus regarding the best criteria for determining sleep quality [2]. Furthermore, various factors can impact sleep quality, and non-restorative sleep is widely acknowledged as one of the most frequently reported reasons for seeking medical consultation [3]. This highlights the need for a clearer understanding of what constitutes good sleep and the mechanisms underlying sleep disturbances.

It is anticipated that the evaluation of sleep quality will emerge as a significant aspect of medical diagnosis in the near future. However, as a multifaceted construct, the natural complexity of sleep makes it difficult to capture its processes using a single measure [4]. Thus, it is necessary to adopt a multivariable approach that incorporates a diverse range of predictors, considering variations in sleep quality that include age and gender information. Previous studies have reported that metrics based on the duration, intensity, or uninterrupted nature of sleep (continuity) have a limited correlation with subjective assessments of sleep quality from the previous night [5]. Alternatively, stability-based measures may have greater significance for future medical diagnoses of sleep quality [2].

In light of these findings, the analysis of sleep microstructure emerges as a crucial aspect in evaluating sleep quality. One particularly significant piece of this analysis is the identification of the Electroencephalogram (EEG) Cyclic Alternating Pattern (CAP) [6], which plays a central role in assessing sleep microstructure. CAP refers to a repeating pattern of changes in brain activity that occurs during sleep and is associated with various markers of sleep quality, including sleep fragmentation and instability. The CAP cycles are composed of alternating activation (A-phase) and quiescent (B-phase) phases that last from 2 to 60 s. The A-phase is characterized by a sequence of transient EEG variations, while the B-phase indicates the recovery of background EEG activity. Additionally, the A-phase can be further classified into three subtypes that play different roles in the sleep process, having distinct amplitude and spectral characteristics in the EEG signal. The first, named A1, is characterized by high-amplitude slow waves, while the third, denoted as A3, is the opposite. The second, entitled A2, represents an intermediate state between the two subtypes [6].

Research has demonstrated that pathological conditions can alter the characteristics of the subtypes, highlighting the importance of examining the CAP pattern and subtype characteristics in assessing sleep quality. Such analysis can provide valuable insights into the stability and fragmentation of sleep and help to identify markers of sleep disturbances, enabling the development of effective strategies for promoting good sleep health.

It is imperative to observe that the division of sleep into a limited number of sleep stages, despite its simplicity, is based on possible obsolescent knowledge about the sleep process [7]. As a result, the metrics estimated based on sleep macrostructure can be considered a rough estimate of sleep quality, as they are based on a synthetic segmentation of the continuous sleep process. Sleep microstructure provides a much more in-depth understanding of sleep, as it is based on a second-by-second analysis of transient and phasic events [8]. However, this increased resolution also brings the challenge of augmented complexity in the analysis, requiring a longer duration for a human operator to perform a full-night sleep examination. To overcome this challenge, it is crucial to automate the examination process to make sleep assessment based on sleep microstructure metrics a feasible possibility [9]. As a result, a fundamental uncertainty is whether automatic CAP analysis is viable. Hence, the formulated research question was: is automatic CAP analysis viable for clinical application?

The goal of this research is to address this query, considering that the examination of CAP, along with other measures of sleep microstructure, can provide a more comprehensive understanding of sleep, enabling the identification of sleep disturbances and the development of effective interventions for promoting good sleep health [10]. A review article was published on automatic CAP methods analysis [11], discussing the performance of automated tools for CAP analysis. Although highly relevant, that review is limited to the performance analysis. Contrarily, this article presents a comprehensive study that not only evaluates the performance of automated tools for CAP analysis but also extends its scope to survey additional articles, encompassing clinical applications and aspects of interpretability. By examining research trends, utilized features, and models, this article aims to find an answer to the formulated research question.

Whilst a deconstruction of arousal circuitries in the human brain is in its infancy, with its cortical and subcortical sources remaining elusive [12–14], the CAP phenotype may provide an indirect fundamental biomarker of its activity [14–17]. Moreover, there is growing evidence that CAP and arousals underwrite the basic mechanisms of sleep regulation, with subtype A1 contributing to the build-up and consolidation of deep slow-wave sleep (SWS), whilst subtypes A2 and A3 contribute to the onset of rapid eye movement (REM) sleep or wakefulness [15], which is also in keeping with findings from recent animal studies [18].

Therefore, for future clinical approach, it might be beneficial in some instances to target various subtypes of CAP, for instance, via new neuromodulation technologies or pharmacotherapy [14]. Moreover, it is likely that ability to record a baseline (untreated) EEG CAP phenotype in majority of sleep or neuropsychiatric disorder would enable a more individualized approach to be developed. For instance, in past, it has been shown that cognitive reserve, daytime sleepiness, affective/mood symptoms and OSA-severity may all dictate the distinct CAP profile in individual patients [14, 17, 19]. Thus, the baseline (untreated) CAP profile may also shape any individualized response to the future treatment in those disorders.

In this background, a systematic literature review was performed to examine the various methodologies for automatic CAP analysis. The study aimed to evaluate the prior work in this area and to identify current trends and advancements. Considering the existing research and technology in this area, the review aimed to provide insights into the potential of these methods to transform the way sleep is analyzed and understood. The organization of this article is as follows. Section 2 presents the methods utilized in conducting the systematic literature review. Section 3 examines the studies included in the review, summarizing the methodology employed in each work. Section 4 consists of an analysis of the performance of the methods, and Sect. 5 concludes the article by presenting the main findings and highlighting the research agenda for future investigations.

## Materials and methods

This section aims to provide a comprehensive overview of the process used to search and analyze the articles. This review study followed the 2020 Preferred Reporting Items for Systematic reviews and Meta-Analyses (PRISMA) guidelines [20] to ensure that the examination is reproducible. Therefore, the eligibility criteria used to determine which studies to include or exclude are presented, specifying the data sources, the method of data collection, and the selection process.

### Search procedure

The systematic article search was conducted using three leading databases: Web of Science, PubMed, and the Institute of Electrical and Electronics Engineers (IEEE). These databases were selected as they offer comprehensive coverage of articles from multiple publishers in various fields, thereby providing a thorough search for the intended topic (automatic methods for CAP examination). The Web of Science indexes an extensive collection of articles from multiple domains, while PubMed focuses on biomedical and life sciences. On the other hand, the IEEE database provides specialized coverage of engineering development analysis. The combination of these three databases offers a comprehensive and complementary search.

The database search was carried out on January 21st, 2023, and aimed to identify all relevant articles aligned with the search strings presented in Table 1. The search keywords used in the search string were chosen to reflect the topic of interest, focusing on sleep patterns and the two most common word derivations associated with CAP (“cyclic alternating pattern” and “CAP”), alongside with “A phase”. Additionally, the keywords “automatic” and “classification” were included to emphasize the focus on automatic procedures in the analysis. The number of results for each search string is presented in Table 1, and the total number of articles found in all databases was 1,280. Among these, 635 were duplicates; thus, the total number of unique articles was 645.

**Table 1:** Number of results for each search string in the examined databases

Search string	Number of results		
	Web of Science	PubMed	IEEE
*sleep –* AND *– cyclic alternating pattern –* AND *– automatic*	67	31	15
*sleep –* AND *– cyclic alternating pattern –* AND *– classification*	73	37	18
*sleep –* AND *– A phase –* AND *– classification*	401	19	98
*sleep –* AND *– A phase –* AND *– automatic*	187	20	49
*sleep –* AND *– CAP –* AND *– classification*	74	39	29
*sleep –* AND *– CAP –* AND *– automatic*	65	36	22
Total	867	182	231

### Eligibility criteria

The initial screening of the 645 articles was performed by two scorers, who reviewed the title and abstract of each article to determine its relevance. The inclusion criteria for the articles were: the article must describe an automatic analysis of CAP, including the classification of A phases, A phase subtypes, or CAP cycles, and be written in English. Articles that only classified the onset or offset of the A phase were not considered for inclusion, as such a method does not provide information about the entire A phase length. After this screening process, a total of 56 articles were selected for further examination.

**Fig. 1:**
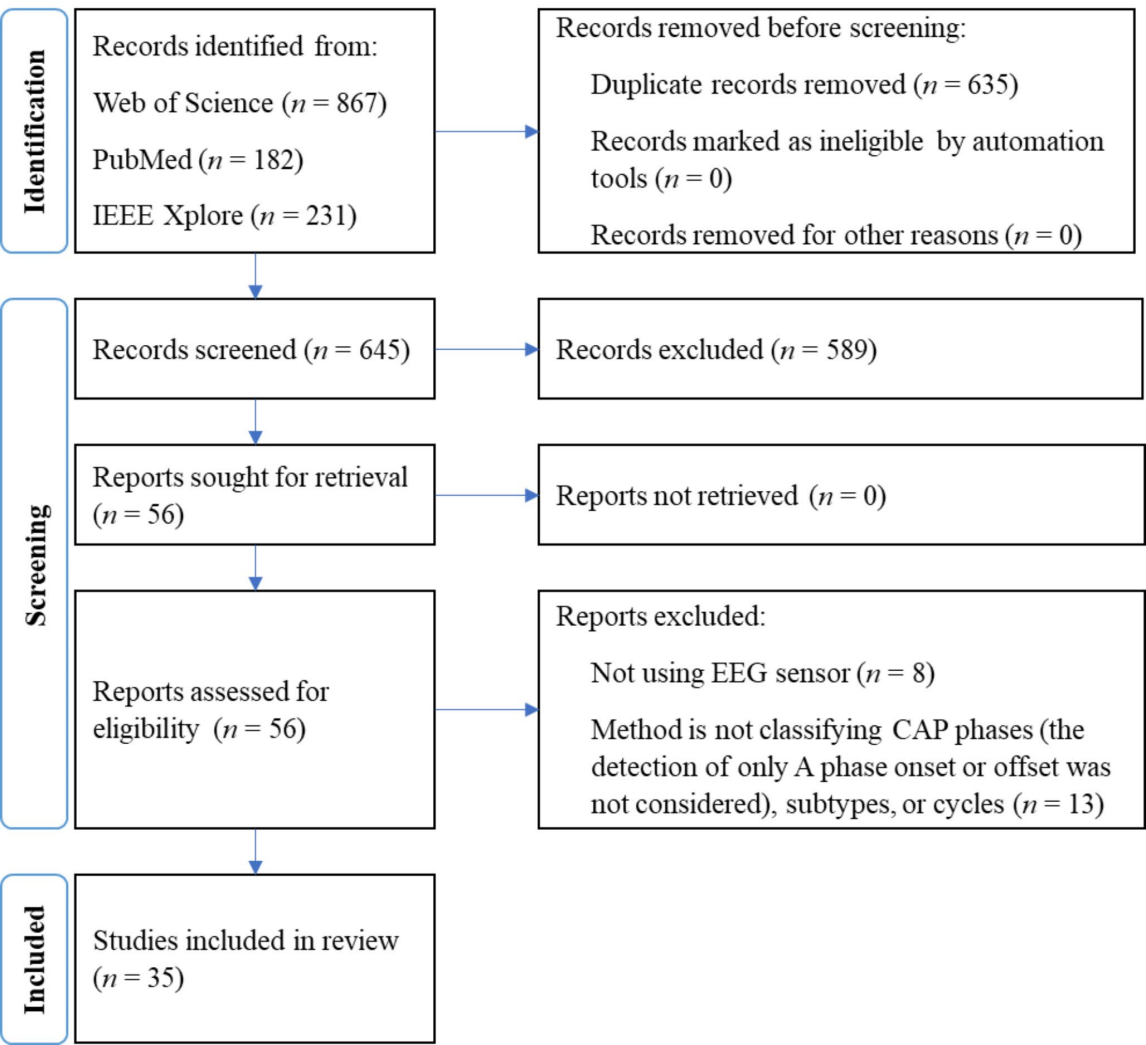
PRIMA flow diagram of the performed systematic review

Eight articles whose method does not examine the EEG sensor were not considered for the review as they employ an indirect analysis regarding the presence of CAP [21–28]. Articles that examined CAP’s signal characteristics but did not provide a fully automatic methodology for A phase, A phase subtypes, or CAP cycle classification were also excluded. As a result, 35 studies were selected for the systematic review. The PRISMA procedure is depicted in Fig. 1.

**Fig. 2:**
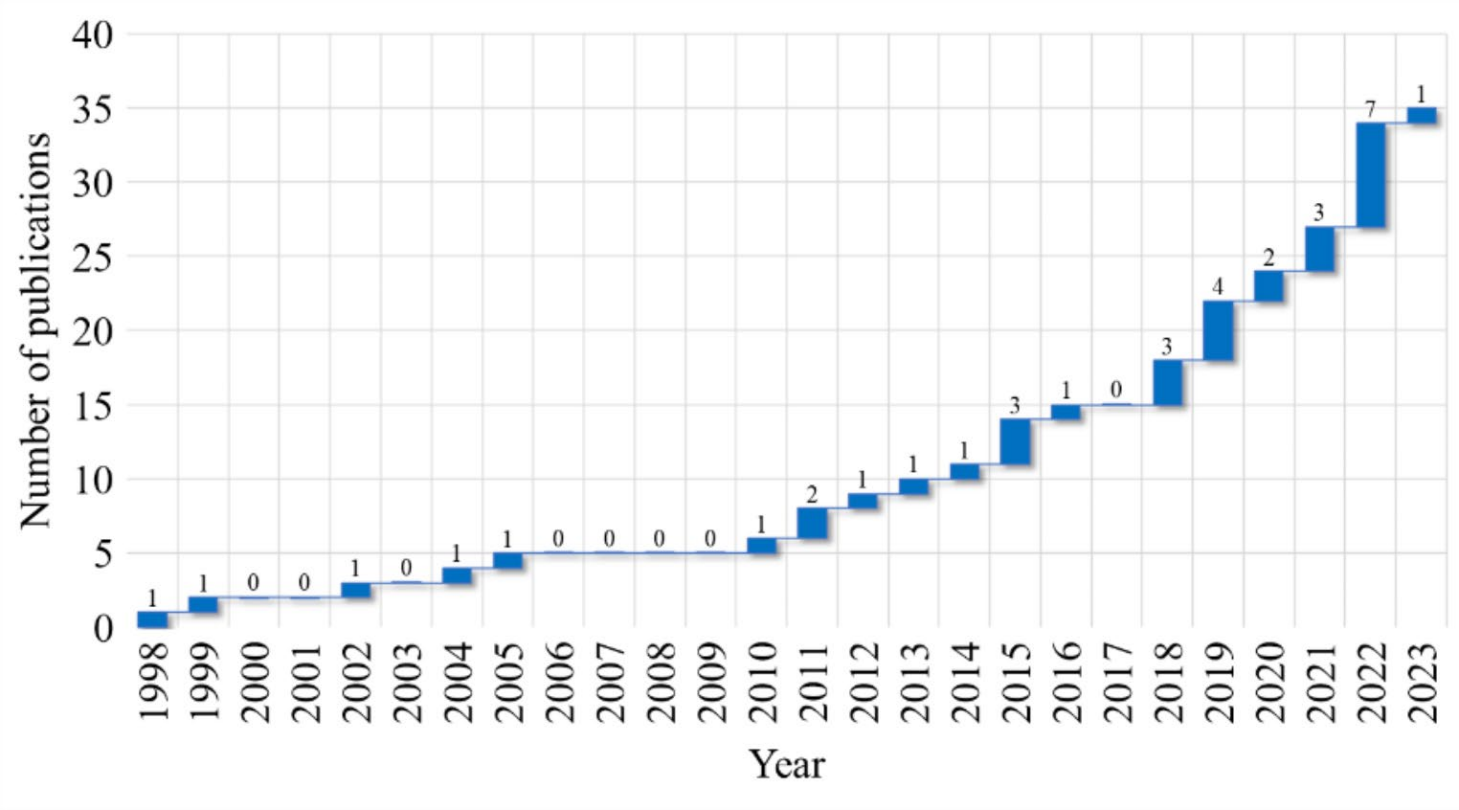
Distribution of the selected articles based on their year of publication, highlighting the accumulated number of publications

The distribution of the selected articles based on their year of publication is presented in Fig. 2. From this figure, it is evident that the search for methods for automatic analysis had already lasted for over two decades. It is also noticeable that there was a nearly stagnant period until 2010. However, interest was resurgent after that, largely due to the advancements in machine learning algorithms and the ability to process larger data sets. This tendency was accelerated after the year 2018 as more than half of the articles (20) were published in the past five years, indicating the significance of the topic and the requirement for a comprehensive review that can consolidate the knowledge, highlight the trends, and identify new directions for exploration.

### Performance analysis

As most studies included in the systematic review employ machine concepts and learning algorithms, four standard performance metrics were considered to assess the relevance of the method’s performance since these were previously shown to be suitable for comparing dissimilar works in a review [29]. Specifically, the metrics were Accuracy (Acc), Sensitivity (Sen), Specificity (Spe), and Area Under the receiver operating characteristic Curve (AUC). Other performance metrics reported by the articles were included but not further analyzed.

## Results

This section summarizes the included articles, presenting their methodologies and results. It is divided into three subsections, following the evolution of automatic classification approaches, from threshold-based classifiers to the conventional machine learning models, concluding with the deep learning models. The results are summarized in Tables 2 and 3, and 4 for the A phase classification, A phase subtypes estimation, and CAP cycle detection, respectively. Most works used the CAP sleep database in the examination [6, 30]. Although some works are certain to be using samples from this database, since they have not explicitly mentioned it, it was not indicated highlighted in the table as using data from that database. Likewise, some works report which subjects from the database were used, but without specifying the demographic characteristics. Hence these characteristics were not included in these works.

**Table 2:** Summary of the reviewed A phase classification procedures, showing only the article’s reported results from best-performing models. The table sorted the articles by classification approach (first thresholds, then conventional machine learning, followed by deep learning). When using the same approach, the results were sorted by performance. In the end, the mean and standard deviation of the metrics reported by at least two studies are presented. Abbreviations: bruxism (B), derivation (D), female (F), healthy (H), insomnia (I), male (M), narcolepsy (N), periodic leg movement (PLM), subjects (S), threshold-based (TB), with ages between (WAB), years old (YO)

Article	Population				Classifier		Performance metrics (%)													
	Subjects	Characteristics					Acc		Sen		Spe			AUC				Other		
[32]	41~	S (8 H, 9 with I, 1 with B, 4 with SDB, and 19 with RBD) using F2-F4/Fp2-F4, F4-C4, C4-P4, P4-O2, C4-A1, and F4-A1 D			TB		-		91.2		-			-				Precision: 45.9; F1 score: 59.6, false negative rate: 8.8, false discovery rate: 54.1		
[34]	10*	H S (all M) using F4-C4 D			TB	-		84.0				90.0			-		Correctness: 77.0			
[36]	8^	-			TB		-		84.9		77.6			-				Concordance: 81.1		
[37]	8	H S (4 M and 4 F) using C3-A2 or the C4-A1 D			TB		72.4		51.6		76.4			-				-		
[39]	6~	H S (4 M and 2 F) using C3-A2 or the C4-A1 D			TB		80.5		75.8		81.3			-				-		
[35]	10	H S (5 M and 5 F) using F4-C4 D			TB		83.5		-		-			-				-		
[38]	16~	H S using C3-A2 or the C4-A1 D			TB		86.1		67.0		89.6			-				-		
[46]	6~	H S using C3-A2 or the C4-A1 D			SVM		72.4		76.8		69.2			79.0				F1 score: 69.9		
[48]	6~	H S using C3-A2 or the C4-A1 D			Ensemble of trees		73.4		76.6		71.0			-				-		
[44]	14 ~ WAB 23 and 78 YO	Using C3-A2 or the C4-A1 D			Linear DA		75.0		78.0		74.0			76.0				-		
[51]	30 ~ WAG 14 and 67 YO (mean 31.0)	NFLE S (16 M and 14 F) using C4-A1 D			SVM		76.0		79.0		76.0			-				Weighted accuracy: 78.0		
[45]	14 ~ WAB 23 and 78 YO	S (9 H, 4 with SDB, and 1 with B) using C3-A2 or the C4-A1 D			FFNN		79.0		76.0		80.0			78.0				-		
[41]	4	H S (4 M and 2 F) using C3-A2 or the C4-A1 D			FFNN		81.6		75.7		83.1			-				-		
[42]	4	H S (4 M and 2 F) using C4-A1 and F4-C4 D			SVM		84.1		73.8		85.9			-				Cohen’s kappa: 50.0		
[43]	8	H S (4 M and 4 F) using C3-A2 or the C4-A1 D			Linear DA		84.9		72.5		86.6			-				Cohen’s kappa: 45.0		
[47]	77 ~ WAB 16 and 82 YO (mean 48.0)	S (6 H, 7 with I, 5 with N, 27 with NFLE, 9 with PLM, 22 with RBD, and 1 with SDB) using C4-A1 and F4-C4 D			Ensemble of trees		78.0		74.0		-			86.0				Precision: 80.0, Cohen’s kappa: 56.0		
[59]	6~	H S using C3-A2 or the C4-A1 D			CNN		53.0		92.1		-			-				Precision: 20.1, F1 score: 33.0		
[58]	6~	H S using C3-A2 or the C4-A1 D			CNN		60.6		69.5		59.3			71.1				Precision: 19.8, F1 score: 30.8		
[53]	14~	D (9 H, 4 with SDB, and 1 with B) using C3-A2 or the C4-A1 D			DAE		67.0		55.0		69.0			-				-		
[56]	15 ~ WAB 23 and 42 YO (mean 32.2)	H S (6 M and 9 F) using C3-A2 or the C4-A1 D			LSTM		69.7		51.2		81.1			66.3				-		
[54]	19 ~ WAB 23 and 78 YO (mean 40.6)	S (15 H and 4 with SDB, 10 M and 9 F) using C3-A2 or the C4-A1 D			LSTM		76.0		74.6		76.6			75.2				Positive predictive value: 65.9, negative predictive value: 82.1, diagnostic odds ratio: 9.6		
[60]	9 ~ WAB 23 and 42 YO (mean 32.2)	H S (5 M and 4 F) using C3-A2 or the C4-A1 D			CNN		76.3		-		-			-				-		
[55]	16 ~ WAB 16 and 67 YO (mean 32.9)	S (8 H and 8 with NFLE, 5 M and 11 F) using Fp23-F4, F4-C4, and the C4-A1 D			LSTM	76.5				72.9		77.1			82.4				-	
[57]	16~	H S using C3-A2 or the C4-A1 D			LSTM	82.4				75.3		83.9			-				F1 score: 57.4	
[65]	19~	S (15 H and 4 with SDB, 10 M and 9 F) using C3-A2 or the C4-A1 D			LSTM	83.0				76.5		83.4			88.2				-	
[63]	15~	H S using C3-A2 or the C4-A1 D			LSTM	83.0				76.1		84.2			-				F1 score: 58.2	
[62]	15 ~ WAB 23 and 42 YO (mean 32.4)	H S (6 M and 9 F) using C3-A2 or the C4-A1 D			Ensemble of CNNs	83.3				80.1		83.7			-				Average: 82.4	
[61]	16~	H S using C3-A2 or the C4-A1 D			CNN	92.5				63.6		96.1			79.8				Precision: 64.5, F1 score: 62.4	
Mean ± standard deviation	14.4 ± 11.3 S			TB		80.6 ± 5.1				76.5 ± 12.4			82.7 ± 5.3			-				-
	18.1 ± 22.2 S			Conventional machine learning		78.3 ± 4.3				75.8 ± 2.0			78.2 ± 6.2			79.8 ± 3.8				-
	13.8 ± 4.3 S			Deep learning		75.3 ± 10.6				71.5 ± 11.0			79.4 ± 9.4			77.2 ± 7.2				-

*Used 60 min of data per subject

^Used a total of 16 h of data

~Reccordings from the CAP sleep database [6, 30]

**Table 3:** Summary of the reviewed A phase subtypes classification methodologies, presenting only the results from the best-performing model of each article. The table first shows the threshold-based classifications, followed by conventional machine learning and deep learning classifiers. The results are then further sorted by performance. Abbreviations: A1 phase (A1), A2 phase (A2), A3 phase (A3), derivation (D), female (F), healthy (H), male (M), subjects (S), thresholds (T), with ages between (WAB), years old (YO)

Article	Population		Classification	Performance metrics (%)													
	Subjects	Characteristics		Acc		Sen			Spe		AUC				Other		
[40]	30~	NFLE S (16 M and 14 F)	A1 – not A1 with T	-	80.3					82.9		-		-			
			A2 – not A2 with T	-	80.0					73.2		-		-			
			A3 phase – not A3 phase with T	-	67.6					74.4		-		-			
[34]	10*	H S (all M) using F4-C4 D	A1 – A2 or A3 with T	-		81.0			81.0		-				Correctness: 79.0		
[51]	30 ~ WAB 14 and 67 YO (mean 31.0)	NFLE S (16 M and 14 F) using C4-A1 D	A1 – A2 – A3 – not A phase with SVM	71.0		A1: 58.0, A2: 44.0, A3: 24.0			76.0		-				Weighted accuracy: 51.0		
[50]	30 ~ WAB 14 and 67 YO (mean 31.0)	NFLE S (16 M and 14 F) using C4-A1 D	A1 – A2 – A3 – not A phase with SVM	71.0		A1: 58.0, A2: 44.0, A3: 24.0, not A phase: 76.0			-		-				-		
[49]	5 WAB 25 and 45 YO (mean 32.7)	H S (2 M and 3 F) using C3-A2 or the C4-A1 D	A1 – A2 – A3 with k-NN	82.2		87.1			74.2		-				-		
[60]	9 ~ WAB 23 and 42 YO (mean 32.2)	H S (5 M and 4 F) using C3-A2 or the C4-A1 D	A1 – A2 – A3 with CNN	70.5		-			-		-				-		
[57]	16~	H S using C3-A2 or the C4-A1 D	A1 phase – A2 phase – A3 phase – not A phase with LSTM	81.9		A1: 63.1, A2: 42.3, A3: 70.6, not A phase: 85.5			-		-				A1 F1 score: 46.6, A2 F1 score: 33.0, A3 F1 score: 60.3, not A phase F1 score: 90.2		
[64]	15 ~ WAB 23 and 42 YO (mean 32.2)	H S (6 M and 9 F) using C3-A2 or the C4-A1 D	A1 – not A1 with LSTM	81.9	87.8			81.6				92.1				-	
			A2 – not A2 with LSTM	79.9	81.1			79.6				88.4				-	
			A3 – not A3 with LSTM	84.6	70.4			85.1				85.5				-	
[62]	15 ~ WAB 23 and 42 YO (mean 32.4)	H S (6 M and 9 F) using C3-A2 or the C4-A1 D	A1 – not A1 with CNN ensemble	83.4	89.6		83.0						-				Average: 85.3
			A2 – not A2 with CNN ensemble	82.3	78.2		82.3						-				Average: 80.9
			A3 – not A3 with CNN ensemble	86.1	78.6		86.3						-				Average: 83.7
[61]	16~	H S using C3-A2 or the C4-A1 D	A1 – A2– A3– not A phase with CNN	88.1	A1: 54.7, A2: 29.7, A3: 60.6, not A phase: 92.4			-				-				A1 F1 score: 46.0, A2 F1 score: 29.1, A3 F1 score: 47.0, not A phase F1 score: 93.9	

*Used 60 min of data per subject

~Reccordings from the CAP sleep database [6, 30]

**Table 4:** Summary of the reviewed CAP cycle classification methodologies, presenting the article’s best-performing results. The table sorts the articles by classification approach (first thresholds, then FSM, followed by conventional machine learning) and performance. At the of the table, the mean and standard deviation of the metrics (reported by at least two studies) are presented. Abbreviations: bruxism (B), derivation (D), female (F), healthy (H), male (M), subjects (S), threshold-based (TB), with ages between (WAB), years old (YO)

Article	Population			Classification	Performance metrics (%)								
	Subjects	Characteristics			Acc	Sen		Spe		AUC		Other	
[38]	16~	H S using C3-A2 or the C4-A1 D		TB	-	-		-		-		CAP rate error: 16.9	
[31]	4	H S		TB	88.5	-		-		-		-	
[33]	4	H S (2 M and 2 F) using C4-A1 D		TB	89.8	89.8		95.0		-		-	
[56]	15 ~ WAB 23 and 42 YO (mean 32.2)	H S (6 M and 9 F) using C3-A2 or the C4-A1 D		FSM	67.9	50.1		89.5		70.3		-	
[44]	14 ~ WAB 23 and 78 YO	Using C3-A2 or the C4-A1 D		FSM	75.0	-		-		-		-	
[54]	19 ~ WAB 23 and 78 YO (mean 40.6)	S (15 H and 4 with SDB, 10 M and 9 F) using C3-A2 or the C4-A1 D		FSM	76.3	71.4		84.2		77.8		Positive predictive value: 67.8, negative predictive value: 77.7, diagnostic odds ratio: 13.3	
[65]	19~	S (15 H and 4 with SDB, 10 M and 9 F) using C3-A2 or the C4-A1 D		FSM	77.7	72.5		80.5		-		CAP rate percentage error: 17.2	
[45]	14 ~ WAB 23 and 78 YO	S (9 H, 4 with SDB, and 1 with B) using C3-A2 or the C4-A1 D		FSM	79.0	-		-		-		-	
[53]	14~	S (9 H, 4 with SDB, and 1 with B) using C3-A2 or the C4-A1 D		FFNN	62.0	67.0		59.0		-		-	
[52]	8~	S (4 H and 4 with SDB) using C4-A1 D		SVM	81.0	-		-		-		-	
Mean ± standard deviation	4.0 ± 0.0 S			TB	89.2 ± 0.6		-		-		-		-
	12.0 ± 2.8 S			FSM	77.0 ± 1.4		72.0 ± 0.5		82.4 ± 1.9		-		-
	16.8 ± 2.3 S			Conventional machine learning	71.5 ± 9.5		-		-		-		-

~Reccordings from the CAP sleep database [6, 30]

### Threshold-based methods

EEG exhibits complex patterns and generates substantial data during a full night examination. Several of these patterns are associated with CAP [10] and comprise amplitude and frequency characteristics. A total of 10 studies propose to automate the CAP analysis by relying on custom thresholds to identify the A phases [31–40]. Lima and Rosa [31] proposed a method that relied on an EEG signal model and looked for changes in the squared signal to detect the A phase. Afterward, a Finite State Machine (FSM) was employed for the CAP cycle detection. Rosa et al. [33] also used an FSM for CAP cycle detection, but employed a method based on a matched filter with a variable length and relative amplitude sliding template to detect A phases and then determined the end of these phases using a convolution-based procedure. Nevertheless, modeling EEG signals, which are complex and generate large amounts of data during a full-night examination, present a difficult challenge.

There is a need to identify characteristics in the data that can emphasize patterns while reducing the amount of information. These characteristics are usually named features, and several have been proposed for CAP analysis. Navona et al. [34] adopted this approach, proposing an A phase detection based on the computation descriptors for characteristic EEG bands (delta, 0.75–4 Hz, theta, 4–8 Hz, alpha, 8–12 Hz, sigma, 12–15 Hz, and beta, 15–25 Hz). The descriptors were computed by averaging the amplitude values of two time intervals, a long interval of 64 s and a short interval of 2 s, every 0.5 s. The computation was then given by (short average − long average) / long average, providing a normalized measure to describe how much the instantaneous amplitude differed from the background amplitude. These features were also used by Barcaro et al. [35]. Largo et al. [36] further extend this idea by proposing an activity index that computes two moving averages, one short and the other long, from EEG bands (the standard bands with the delta band in three sub-bands, from 0.5 to 1 Hz, 1–2 Hz, and 2–4 Hz) obtained from a discrete wavelet transform.

Mariani et al. [37] further analyzed the band descriptors (the conventional bands with the delta subdivided into low, 0.5–2 Hz, and high, 2–4 Hz). They also utilized the differential variance of the EEG signal (calculating the difference in variance between consecutive windows) and Hjorth descriptors in the low delta and high delta bands. These Hjorth descriptors were activity (variance of the signal segment) and mobility (the square root of the ratio of the activity of the first derivative of the signal to the activity of the original signal). It was concluded that differential variance provides the highest Acc and Spe. Mariani et al. [38] first segmented the EEG signal using a FeedForward Neural Network (FFNN) to separate the Non-Rapid Eye Movement (NREM) sleep. Then, they used the previously mentioned features (5 band descriptors, Hjorth activity, and differential variance) for A phase analysis and applied the CAP scoring rules to identify the CAP cycles. Machado et al. [40] examined subjects with Nocturnal Frontal Lobe Epilepsy (NFLE) and computed for the five standard EEG bands the bands’ descriptors and the Teager Energy Operator (TEO). It was concluded that the best performance for A1 and A2 subtypes was attained using TEO in the delta bands, while for A3, it was using the beta band.

A different approach was proposed by Fantozzi et al. [32] that studied healthy and sleep disorder subjects, including insomnia, bruxism Sleep-Disordered Breathing (SDB), and REM Behavior Disorder (RBM). They filtered the EEG signal into two bands (slow, 0.3–4.5 Hz, and fast, 7–25 Hz) and then proposed an algorithm that uses the root mean square of the signal to identify the presence of A phases. Niknazar et al. [39] also proposed a conceptually different algorithm based on local extrema’s statistical behaviors to determine the A phases’ start and end times by examining the EEG delta band.

### Methods based on conventional machine learning models

The use of threshold-based methods for CAP signal analysis may seem intuitive, given that these signals exhibit dissimilar amplitude and frequency characteristics. However, it is challenging to generalize a threshold tuned for a specific dataset to a broader population. This difficulty is evident from the trend observed in the year of publication (Fig. 2); except for Fantozzi et al. [32], all other works relying on threshold methods had been published prior to 2015. This likely reflects the generalization problem inherent in threshold-based processes. The following analysis focuses on methodologies that use machine learning algorithms, enabling the models to learn the relevant characteristics from the data. A total of 12 articles compose this examination.

In their study, Mariani et al. [41] suggested using an FFNN fed with the features described by Mariani et al. [38]. However, only the NREM sleep was analyzed. Although it is logical to isolate NREM sleep, manually doing so can hamper the practical applicability of the proposed methodology. It is, therefore, advisable to either keep all sleep data or employ an automatic process to segment the NREM sleep. Another important aspect is the used postprocessing procedure that divided the scored long A phases (over 60 s) into two separate A phases using a neural network-based clustering method. Both preprocessing and postprocessing are critical components in machine learning, as the former prepares the data, while the latter corrects some misclassifications. A Support Vector Machine (SVM) (fed with similar features to those used in the previous study) was employed by Mariani et al. [42], presenting a postprocessing procedure capable of correcting misclassifications by changing isolated 1-second classes to the adjacent class. Later, Mariani et al. [43] adopted a similar approach but examined four classifiers, specifically, FFNN, SVM, adaptive boosting with trees, and linear Discriminant Analysis (DA). Among the four classifiers, the DA achieved the highest accuracy and specificity.

Linear DA classifier was also used by Mendonça et al. [44], which segmented the EEG signal into two-second segments and estimated six time-based features (average power, standard variation, Shannon entropy, autocovariance, log-energy entropy, TEO) and five frequency-based features by examining the Power Spectral Density (PSD) in the delta, theta, alpha, sigma, and beta bands. Sequential Feature Selection (SFS) identified PSD in the beta, theta, and alpha bands, average power, TEO, and standard deviation as the most relevant features. An FSM was also used to assess the CAP cycles. Later, Mendonça et al. [45] expanded the work by examining nine more classifiers, the Logistic Regression (LR), two tree-based methods (one with and one without ensemble), SVM, kNN, two variants of the FFNN, and unsupervised learning-based classifiers, the SelfOrganizing Map (SOM) and the k-Means Clustering (k-MC). It was concluded that the standard FFNN outperformed the other classifiers using the PSD in the theta and beta bands, Shannon entropy, TEO, and autocovariance as features.

Dhok et al. [46] used the Wigner–Ville distribution to analyze two-second segmented data, which enables exhaustive time-frequency analysis. They then calculated the Rényi entropy and fed the result into an SVM to classify the A phase. To ensure balanced performance, they performed a balancing operation. A time-frequency approach was also proposed by Sharma et al. [47], using an orthogonal filter bank and wavelet to decompose the EEG signal into six subbands. Then they computed the wavelet entropy and three Hjorth parameters (activity, mobility, and complexity) from each subband to produce 48 features. Two tree-based classifiers (one with bagging and the other with boosting), SVM, and k-NN were studied for the A phase classification. The tree-based classifier with bagged trees attained the best performance using balanced data (the authors reported individual performance for multiple sleep disorders, however, in Table 2, only the healthy subjects’ results are shown). Sharma et al. [48] also used wavelet decomposition to attain six subbands and computed both the approximate and entropies for each band. An ensemble of boosted trees was then used to classify the occurrence of A phases with a balanced dataset.

Mendez et al. [49] presented a method for further distinguishing A-phase subtypes from previously classified A-phases. For this purpose, two-second segments were analyzed and computed for each segment the mode, standard deviation, skewness, kurtosis, energy, and power after spectral decomposition of the EEG signal in four bands (delta, theta, alpha, and beta). Complexity and entropy measurements (Lempel-Ziv Complexity, Sample Entropy, Fractal Dimension, and Tsallis Entropy) were also computed in sliding windows of 4 s with 2 s of overlap. The classification was then carried out using the k-Nearest Neighbors (k-NN) algorithm. Machado et al. [50] further expanded this concept by creating a methodology for identifying the subtypes of the A phase directly from the EEG signal. They utilized the EEG band descriptors (previously described), TEO, zero-crossing, Lempel-Ziv complexity, characteristics of the discrete-time short-time Fourier transform signal (such as frequency of maximum and mean energy and area under the magnitude spectrum curve), empirical mode decomposition, Shannon entropy, fractal dimension, and variance of the EEG signal. A total of 55 features were created, and the minimum Redundancy Maximum Relevance (mRMR) algorithm was used to rank them. The top 40 ranked were fed into an SVM (k-NN and linear DA were also examined but attained a lower Acc). Later, Machado et al. [51] used the same methodology but provided results for A phase detection and examined the use of Principal Component Analysis (PCA) to reduce the features’ dimensionality. However, the results without PCA were superior. The same classifiers were examined by Karimzadeh [52] for CAP cycle detection. An SFS procedure was then used to determine the most relevant features, choosing Kolmogorov, Shannon, and Sample Entropy to feed an SVM (best-performing classifier).

### Methods based on deep learning models

Despite being intuitive, relying on features designed by researchers has significant drawbacks in the context of analyzing CAP patterns. Feature engineering is a demanding process that requires expertise and thoughtful consideration, often involving a feature selection procedure to identify the most relevant features for the problem at hand. This process can be time-consuming and may not always result in optimal features. Additionally, the features are limited in their ability to capture complex patterns and relationships in the data, leading to poor generalization and potential scalability issues as the amount of data increases. In contrast, deep learning-based methods can automatically learn relevant features from the data, uncovering patterns that may not be immediately apparent to humans. This eliminates the need for manual feature engineering and allows for the effective handling of large amounts of data. A total of 13 articles employed deep learning classifiers.

Mostafa et al. [53] propose the first deep learning model for A phase analysis (in 2018), using a Deep AutoEncoder (DAE), whose output was then stored in a buffer to feed a subsequent FFNN responsible for classifying the CAP cycles. Mendonça et al. [54] provided the preprocessed EEG signal to three classifiers, two are based on a Recurrent Neural Network (RNN), precisely, the Long Short-Term Memory (LSTM) and the Gated Recurrent Unit (GRU). The last model was a Convolutional Neural Network (CNN) with one-dimensional input and custom architecture. The result of the A phase classification was then fed to an FSM to classify the CAP cycles. It was reported that LSTM attained the utmost performance. Mendonça et al. [55, 56] followed a similar methodology with an LSTM, which was also the classifier employed by Hartmann and Baumert [57] (as a comparison, an FFNN was used, which achieved lower performance). They propose cleaning procedures to remove cardiac field and eye movement artifacts. Furthermore, a balancing process was employed to balance the data. The network structure was optimized by a genetic algorithm and particle swarm optimization, reaching the best performance using three EEG derivations as input. The LSTM layers performed the information fusion and provided the result to dense layers to classify the A phases.

Murarka et al. [58] presented a CNN architecture with one-dimensional input and employed an undersample balancing technique. The results in Table 2, however, show the unbalanced data performance to enable comparison with other deep learning studies. The authors evaluated the individual performance for various sleep disorders, but Table 2 only displays the results for healthy subjects. Loh et al. [59] adopted a similar approach by proposing a CNN architecture and using a balancing method. Therefore, Table 2 presents the unbalanced data performance (for the same reason as before).

Arce-Santana et al. [60] proposed another CNN architecture fed with spectrograms, which in this work are two-dimensional representations of four-second segments of the EEG signal. The authors followed a training procedure where the network was first trained using 12.5% of the subject’s data and then used to classify the remaining 87.5% segments. Afterward, the network was retrained with 20–50% of the data classified by a specialist. To allow for comparison with other deep learning studies, Tables 2 and 3 include results without the retraining procedure. The proposed algorithm is capable of classifying the A phase and its subtypes. A methodology with the same classification capability was presented by You et al. [61], proposing an encoder-decoder CNN architecture based on the U-net framework (with skip connections) with a transformer layer incorporating a gated multi-head attention mechanism. The article reports performance for healthy and subjects NFLE subjects. However, Tables 2 and 3 only comprise the results related to the entire sleep data of the healthy subjects.

Mendonça et al. [62] put forward a method that employs long windows of EEG signals with overlapping durations (ranging from 15 to 23 s) as inputs for an ensemble of three CNNs. Each CNN has a one-dimensional input and is optimized separately using the HOSA algorithm. The first CNN is fed with data that overlap to the right, and the second CNN receives inputs that overlap to both the left and right. The third CNN uses data that overlaps to the left. The output from the three classifiers was combined to classify the A phase or its subtypes. Additionally, they introduce the A-phase index as a complementary perspective for CAP analysis, which provides a visual representation of sleep stability. The study involved healthy subjects and individuals with sleep disorders (NFLE, insomnia, and SDB), but the results in Tables 2 and 3 pertain solely to the healthy subjects.

Deep learning models can also incorporate features as input. Hartmann and Baumert [63] explored this possibility using Hjorth activity, Shannon entropy, TEO, differential EEG variance, and band descriptors. These features were fed to three conventional machine learning models (linear DA, k-NN, and FFNN) and an LSTM that outperformed the other classifiers in A phase classification. Mendonça et al. [64] compared the performance of deep learning models fed with features against the same model provided directly with the preprocessed EEG signal. The features analyzed three main aspects of the EEG signal: amplitude through symbolic dynamics and an amplitude variation metric; frequency through PSD of the five characteristic EEG frequency bands; and the ratio of the maximum amplitude of an epoch to its calculated PSD, which represented both amplitude and frequency. The relevance of the features was measured using mRMR, and the most important were employed for the A phase subtype classification. The results indicated that using features improved performance, likely because the limited data did not allow the deep learning model to learn all relevant characteristics. These features were later used by Mendonça et al. [65] that conducted a similar analysis but proposed the Heuristic Oriented Search Algorithm (HOSA) for optimizing the structure of deep learning models. The authors examined the performance of LSTM fead with features agains the LSTM fead with the preprocessed EEG signal, and concluded again that the use of the feature-based model was superior for the same reason as previously stated. They also tested a FFNN and a CNN, and performed CAP cycle detection using a FSM.

## Discussion

This section examines and discusses the reported results of the surveyed works. The performed classification was first explored, followed by an overview of the used features and classifiers and their relation to the CAP analysis.

### Reviewed works’ performance

Out of the 35 articles reviewed, as observed in Tables 2 and 28 performed binary classification of EEG epochs as either A phase or not A phase, with seven using a threshold-based classifier, nine using conventional machine learning classifiers, and 12 using a deep learning classifier. Additionally, ten articles examined the A phase subtypes. Among them, two used a threshold-based classifier, three used conventional machine learning classifiers, and five used a deep learning classifier.

Various approaches were employed for subtype detection, shown in Table 3, including multiclass models, individual models for each subtype, and models that separate previously classified A phases. This diversity of methodologies makes it impractical to compare the results. Furthermore, six studies conducted both A-phase and A-phase subtype analyses, [34, 51, 57, 60–62], while the remaining studies, [40, 49, 50, 64], only performed A-phase subtype classification.

Lastly, ten articles examined the CAP cycles, presented in Table 4, with three using a threshold-based classifier, two using conventional machine learning classifiers, and five employing an FSM to implement the CAP scoring rules for scoring the CAP cycles. It is worth noticing that no work employed a deep learning model for directly classifying the CAP cycles. Furthermore, most methodologies used for CAP cycle detection rely on a prior A phase classifier whose output was fed an FSM for imposing the CAP cycle rules. Only three works, [31, 33, 52], directly classified the CAP cycles without first estimating the A phases.

**Fig. 3:**
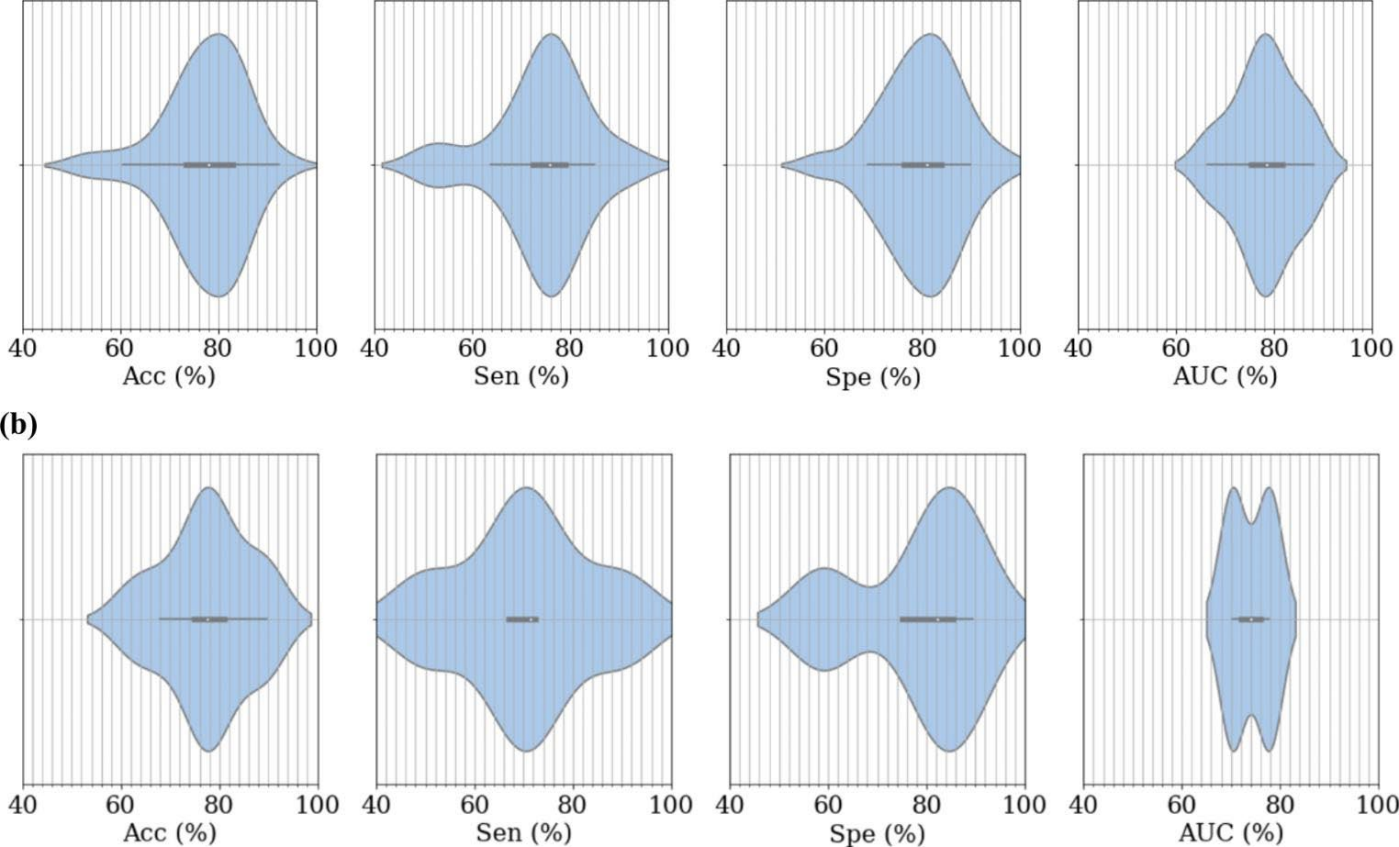
Visualization of the performance metrics distribution for **a** A phase and **b** CAP cycle detection

The follow-up analysis focused solely on A phase and CAP cycle detection, aiming to evaluate the current state-of-the-art classification performance, regardless of the methodology used, as a possible alternative to manual scoring. Violin plots with the results for the main examined performance metrics are shown in Fig. 3. It was reported that sleep specialists’ agreement to score CAP events could range from 69 to 78% [66]. By checking the distributions from Fig. 3, it is notorious that the median is around 78%, which is precisely the upper specialist agreement. Although in a crude examination, it can be inferred that the current automatic models are as good as specialist scoring CAP, supporting the viability of automation for CAP examination. It is also worth noting that most works used the same dataset and examined the same subjects, making this analysis less subjective.

The spread in performance can be attributed to the substantially different methodologies employed. However, it is worth noting that methods that require manual isolation of A phases or consider only data from NREM sleep may enhance model performance but prove impractical for real-world applications. It is also crucial to ensure subject-independent results to avoid bias, particularly when the number of subjects is low. Furthermore, the AUC suggests that reported performance is reasonably balanced, with similar sensitivity and specificity. This is significant because CAP analysis is naturally unbalanced, with far fewer data relating to the A phases than to not A phase. As a result, a high Acc is ambiguous without reporting sensitivity and specificity. Similarly, if a balancing operation is conducted, the test data should remain unchanged, as modifying the natural data distribution makes it impossible to ascertain whether the reported results will generalize to new, unseen data.

Regarding the A phase classification performance, the highest Acc (92.5%) and Spe (96.1%) were reported by You et al. [61], but their Sen was too low (63.6%), which is aggravated by the inherent imbalance in A phase analysis and limits the method’s practicality. In contrast, Loh et al. [59] reported the highest sensitivity (92.1%), but their accuracy (53.0%) was nearly at a random level, rendering the approach unreliable. Therefore, the method proposed by Mendonça et al. [62] is likely the most suitable for clinical application since it reported the best-performing balanced results (Acc, Sen, and Spe of 83.3%, 80.1%, and 83.7%, respectively) and did not require any manual manipulation of the EEG signals (such as isolating NREM sleep). It is worth mentioning that all three of the indicated studies employed a CNN-based classifier, providing evidence for the suitability of deep learning models in A phase analysis. As for CAP cycle detection, Rosa et al. [33] method achieved the highest performance with an accuracy, sensitivity, and specificity of 89.8%, 89.8%, and 95.0%, respectively. However, since the study only evaluated four subjects, the generalizability of the results may be limited.

### Overview of the used methodologies

The patterns contained within the CAP phases comprise characteristics from the signal’s amplitude and frequency. As a result, most feature-based studies tend to examine features that explore these domains. These features were categorized into three groups: amplitude-based (which assess variations in the signal amplitude), frequency-based features (which examine characteristics in the frequency domain, such as the PSD), and amplitude-frequency-based features (for example, the ratio of maximum amplitude to the calculated PSD of an epoch). However, some features do not fit into these categories, so three additional were included: statistics-based features (such as mean or kurtosis), entropy-based features (such as Shannon entropy), and complexity-based features (which explore the signal complexity without relying on entropy). Additionally, Hjorth parameters were included as a separate category since these comprise different metrics, and as some studies did not identify which one was used, it was impossible to classify them into the previous six categories. It should be noted that certain features may fit into multiple categories, but each feature was only associated with one class to simplify the examination.

**Fig. 4:**
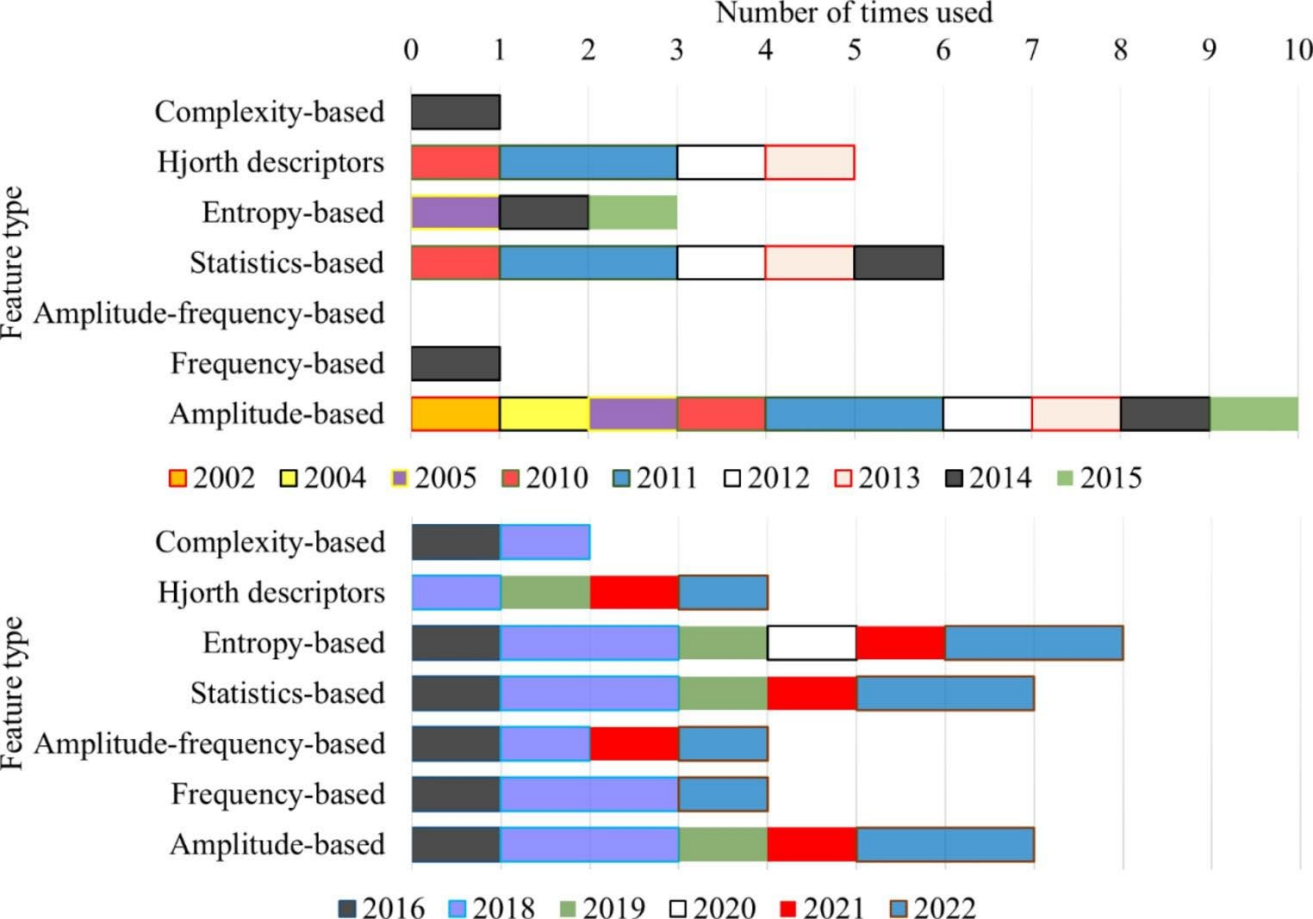
Number of times each feature category was used by a study (and the year of publication of that study)

The number of times each feature-based category was used, and the year of publication of the study that used it, is presented in Fig. 4. Upon examining the figure, it is evident that amplitude-based features were the most frequently used and were reported in studies published throughout the analyzed period. This suggests a strong preference for using these features, possibly due to the predominance of A1 phase subtype characteristics in healthy subjects and the strong association between this subtype and EEG amplitude variations. While other categories of features can also examine these properties, it is noteworthy that frequency-based features were used less frequently, despite the strong connotation between frequency components and CAP. Entropy-based, complexity-based, and Hjorth descriptors features may also be suitable for CAP examination, as they can detect the complex and variable patterns of brain activity during the A phases.

**Fig. 5:**
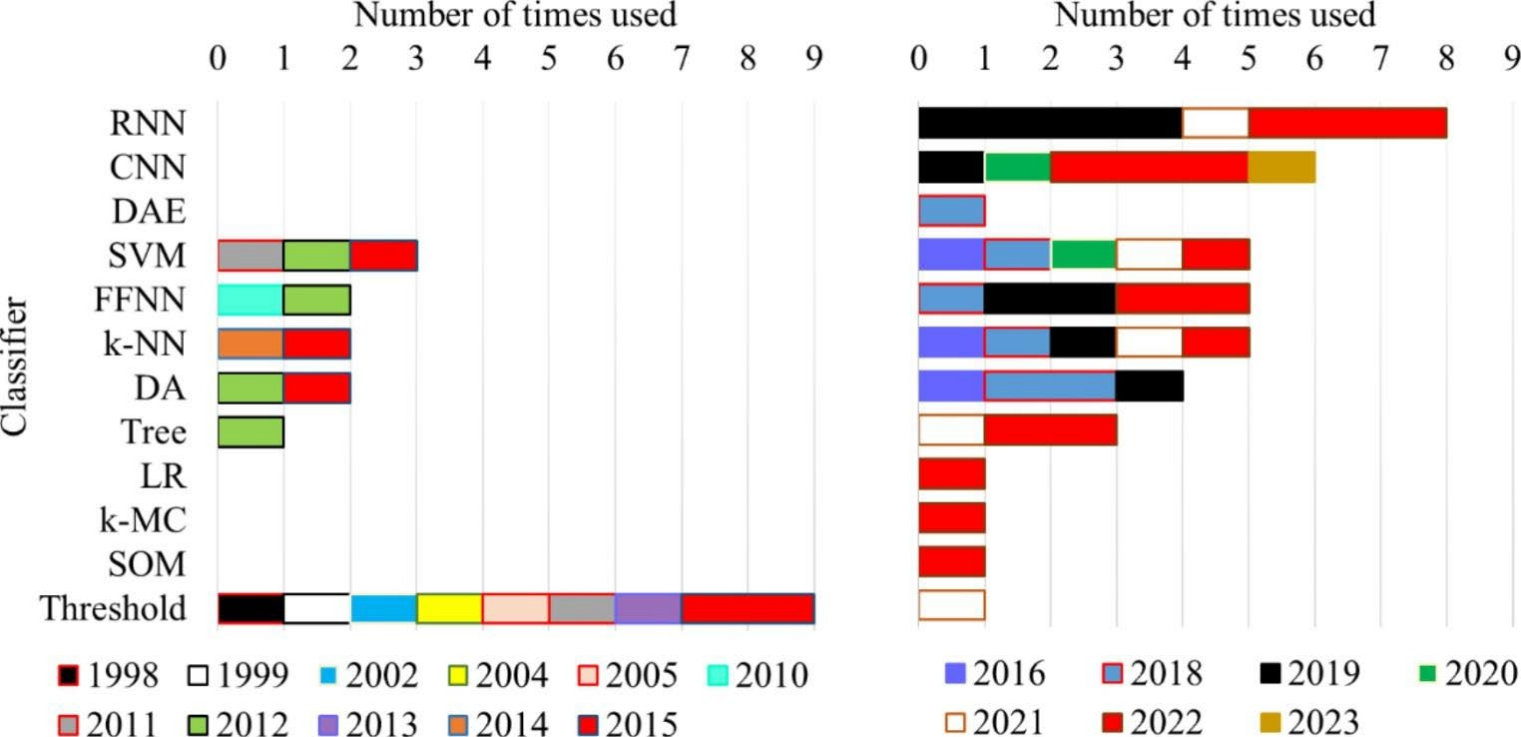
Number of times each classifier was used by a study (and the year of publication of that study)

The subsequent examination is related to the classifiers used by the reviewed works. The distribution of the classifiers by the year of publication of the study and the number of times each classifier was used are presented in Fig. 5. The majority of the classification methodologies used a threshold solution. However, it is important to note that except for one study published in 2021, all other articles that used this methodology were published up to 2015. In contrast, neural network-based methods have been more prevalent in the past four years, primarily due to the growing popularity of deep learning-based approaches. The fact that the best results for A phase analysis were obtained using deep learning models, combined with the continuous growth of available data, suggests that the trend of using neural networks is likely to persist, further reducing the use of conventional machine learning models.

## Conclusion

This study aimed to determine whether automatic CAP analysis is currently achievable. A systematic review was performed to address this question by searching three prominent databases: a standard indexing database, a database dedicated to medical publications, and a database focused on engineering applications. A total of 35 articles were reviewed (from the 1,280 articles initially found), published between 1998 and January 21st, 2023. These studies proposed various methods for automatically examining CAP, including the classification of A-phase, their subtypes, or the CAP cycles.

It was observed that three main trends were used over time regarding the A phase classification. Initially, either mathematical models or features were utilized and classified with a tuned threshold. This trend was followed by the adoption of conventional machine learning models, which have been the norm until the last five years, when there has been a surge in the application of deep learning models. Regarding the classification of CAP cycles, most studies employed an FSM-based approach after A-phase classification to implement the CAP scoring rules. As such, these methods depend on an initial A-phase classifier. Furthermore, the assessment of the A phase subtypes’ performance has proven challenging due to the use of various approaches, ranging from classification with a multiclass model to using individual models for each subtype.

While the current studies have methodological limitations, the performance results determined in this review are consistent and can be considered a reasonable estimate. Notably, the median accuracy of the state-of-the-art methods was comparable to the upper limit of the specialist agreement range, suggesting that automatic CAP analysis can be reliably performed. Therefore, this study provides a positive answer to the main research question.

The recommended research agenda involves validating the proposed methodologies on larger datasets, including more subjects with sleep-related disorders, providing the source code for independent confirmation of the proposed methods, and exploring the possibility of including CAP analysis as a standard sleep examination practice in the future.
